# Molecular Dissection of Permanent vs. Reperfused Ischemia: Multi-Omics Divergence and Precision Therapeutic Implications

**DOI:** 10.3390/cimb48010124

**Published:** 2026-01-22

**Authors:** Zhiyong Shen, Yuxian Li, Tengfei Zhu, Ting Yang, Shiyu Zhou, Qian Liu, Qiong Lu, Dongyan Jing, Haiou Jiang, Jie Li, Xiao-Liang Xing

**Affiliations:** 1 School of Basic Medical Sciences, Hunan University of Medicine, No. 492, Jinxi South Road, Huaihua 418000, China; 2 School of Medical Information and Engineering, Hunan University of Medicine, No. 492, Jinxi South Road, Huaihua 418000, China; 3 MOE Key Laboratory of Pediatric Rare Diseases, University of South China, No. 28, Changsheng West Road, Hengyang 421009, China; 4 Huaihua Key Laboratory of Ion Channels and Complex Diseases, Chengdong Area, Huaihua 418000, China; 5 School of Public Health and Emergency Management, Hunan University of Medicine, No. 492, Jinxi South Road, Huaihua 418000, China; 6 School of Medical Laboratory Science, Hunan University of Medicine, No. 492, Jinxi South Road, Huaihua 418000, China

**Keywords:** permanent ischemia, ischemia reperfusion injury, multi-omics, proteomic remodeling, post-transcriptional regulation

## Abstract

Objective: Cerebral ischemia–reperfusion injury (IRI) is a distinct pathological phase that differs from permanent ischemia (IR) in that it triggers secondary damage despite the restoration of blood flow. The primary objective of this study is to comprehensively characterize and compare the molecular signatures—such as differential gene expression, protein activation, and metabolic alterations—between IRI and IR. By doing so, we aim to identify key pathways and biomarkers that specifically drive IRI and IR pathology, thereby providing novel therapeutic targets to mitigate reperfusion-induced damage in stroke and related neurological conditions. Methods: We employed an integrated transcriptomic and proteomic approach to compare a permanent ischemia model (IR, 24 h ischemia) with a reperfusion model (IRI, 1 h ischemia + 24 h reperfusion), using SHAM-operated animals as controls. Results: Our results demonstrate a profound decoupling between the transcriptome and proteome in IRI. While IRI induced extensive proteomic alterations (160 changed proteins in IRI vs. IR), transcriptional changes were minimal (3 genes), indicating dominant post-transcriptional regulation. Both IR and IRI activated shared inflammatory responses (e.g., Saa3, upregulated 14.33-fold in IRI/SHAM) and metabolic shifts (Gapdh, downregulated 4.03-fold). However, IRI uniquely upregulated neuroprotective genes (Arc, Npas4), activated a specific set of reperfusion-related pathways (72 proteins), and exhibited distinct extracellular matrix remodeling (Mmp3, upregulated 11.24-fold in IR/SHAM). The overall correlation between transcriptomic and proteomic dynamics was remarkably low (r = 0.014), underscoring the importance of translation and protein decay mechanisms. Conclusions: This study redefines IRI not merely as an exacerbation of ischemic damage but as a unique adaptive molecular trajectory. We identify Pisd-ps3 and Saa3 as potential therapeutic targets and show that proteomic signatures can stratify injury phases. These findings advance the prospects of precision therapeutics aimed at neuroprotection and immunomodulation in ischemic stroke.

## 1. Introduction

Ischemic stroke, constituting over 80% of cerebrovascular events, represents a major global health challenge due to its associated high mortality and long-term disability [1,2]. While revascularization therapies, such as thrombolysis and thrombectomy, have improved clinical outcomes, more than half of patients still experience futile recanalization—neurological deterioration despite successful vessel reopening. This underscores a critical pathophysiological divide between permanent ischemia (IR) and ischemia–reperfusion injury (IRI) [3,4]. IR is primarily driven by sustained metabolic failure, activating pathways like RIPK1-RIPK3-MLKL-mediated necroptosis. In contrast, IRI entails a biphasic insult: initial hypoxia–ischemia followed by a reperfusion phase characterized by oxidative stress, intense inflammation, blood–brain barrier breakdown [5], and ferroptosis [6,7]. This mechanistic divergence is further complicated by patient-specific factors, such as age and comorbidities, which can attenuate therapeutic efficacy, highlighting the urgent need for stratified treatment strategies beyond a one-size-fits-all approach.

Multi-omics technologies have profoundly advanced our spatiotemporal understanding of stroke pathology. Spatial transcriptomics has unveiled critical intercellular communication axes, such as LGALS9-CD44 in microglia–oligodendrocyte interactions, which support recovery [8]. Integrated single-cell and metabolomic studies reveal cell-type-specific vulnerabilities; for instance, endothelial ferroptosis via SFXN4 upregulation and oligodendrocytic metabolic dysregulation in IRI [9]. Large-scale genetic analyses have further identified risk loci with opposing roles in IR versus IRI contexts, such as genes within the PI3K-Akt pathway [10,11,12]. These approaches also delineate injury timelines, showing that IRI exhibits an accelerated oxidative stress peak and more sustained proteomic alterations compared to IR.

Despite these insights, translating multi-omics findings into therapies remains challenging. Interventions such as SOCS-1 activation for IR or PPARα agonists for IRI show pathway-specific promise [13,14], and stem cell therapies demonstrate multimodal regulatory potential [15]. However, gaps persist in data integration, and existing models often fail to fully recapitulate the clinical complexity of aging and comorbidities [16]. A pivotal yet underexplored question is the fundamental molecular distinction between IR and IRI at a systems level. Current understanding often views IRI merely as an exacerbation of ischemic damage, lacking a precise characterization of its unique molecular trajectory, particularly regarding the concordance—or profound discordance—between transcriptional and proteomic landscapes.

This study directly addresses this gap by employing integrated transcriptomics and proteomics to systematically compare permanent cerebral ischemia with ischemia–reperfusion. We hypothesize that IRI represents a distinct molecular state, governed significantly by post-transcriptional regulation, and entails unique adaptive and maladaptive pathways not seen in IR. Our analysis confirms a striking decoupling between transcriptome and proteome during reperfusion, identifies reperfusion-specific molecular signatures, including activated neuroprotective programs, and pinpoints key candidates, like Saa3 and Pisd-ps3, for targeted intervention. By redefining IRI through its unique multi-omics footprint, this work provides a refined framework for developing phase-specific, precision therapeutics in ischemic stroke.

## 2. Materials and Methods

### 2.1. Mouse Model of Cerebral Ischemia

The middle cerebral artery (MCA) is the most frequent site of stroke occurrence, with cerebral ischemia classified into two primary types: permanent occlusion cerebral ischemia and ischemia–reperfusion cerebral ischemia. The middle cerebral artery occlusion (MCAO) animal model, established via intraluminal suture embolization, is a globally recognized standard for studying focal cerebral ischemia. To induce cerebral ischemia in mice, the thread embolism method was employed. Following abdominal anesthesia, the mice were positioned supine, and the right common carotid artery (CCA), external carotid artery (ECA), and internal carotid artery (ICA) were carefully dissected and exposed. The proximal ends of the CCA and ECA were ligated, and an MCAO suture (1622, Xinong, Beijing, China) was advanced from the CCA incision into the ICA to occlude the MCA. Permanent occlusion model: The suture remained in place for 24 h without removal, inducing sustained ischemia. Ischemia–reperfusion model: After 60 min of occlusion, the suture was gently withdrawn to restore blood flow, creating 1 h of ischemia followed by 24 h of reperfusion. Post-model preparation, the mice were incubated in a warm environment to facilitate recovery from anesthesia, thereby improving the success rate of the experimental model. In this study, male C57BL/6 mice (Hunan SJA Laboratory Animal Co., Ltd., Changsha, China) aged 8–10 weeks and weighing 20–23 g were obtained. The animals were housed in a pathogen-free animal facility with 12 h light–dark cycles.

### 2.2. Neurological Score Assessment

The neurological deficit score (NDS), according to a 5-point Bederson scale, was employed to systematically evaluate the functional status of the model mice (Supplementary Table S1, Animal behavior evaluation of cerebral ischemia). The scoring criteria were as follows: a score of 0 indicated that the mouse exhibited essentially normal activity without any discernible neurological symptoms; a score of 1 was assigned when the mouse was unable to fully extend its left front paw; a score of 2 denoted a tendency to turn to the left while crawling; a score of 3 signified that the mouse would dump onto the hemiplegic side while walking; and a score of 4 was given when the mouse was unable to walk autonomously and displayed signs of conscious disturbance. We excluded individuals without nerve damage after surgery or with excessive nerve damage who died within 24 h after surgery to avoid the deviation caused by non-research factors. Only those mice with nerve injury disorder of cerebral ischemia were included in the experimental group to ensure a focused and relevant analysis.

### 2.3. Real-Time Monitoring of Regional Cerebral Blood Flow in the Middle Cerebral Artery (MCA) Territory

During the establishment of the cerebral ischemia model in the mice, a precise surgical procedure was carried out. A parietal incision was made to expose the skull, after which the periosteum was meticulously scraped away to ensure a clear and unobstructed surgical field. Subsequently, a specialized probe was securely fixed in place. This probe enabled real-time monitoring of the blood flow distribution across the entire brain, with a particular focus on the region supplied by the middle cerebral artery (MCA) [17,18]. This approach allowed for continuous and dynamic assessment of cerebral perfusion during the critical phase of model construction.

### 2.4. RNA Extraction

The mice were euthanized via cervical dislocation. Immediately following euthanasia, the cortical brain tissue was swiftly harvested and snap-frozen in liquid nitrogen. The frozen tissue was then transferred to a pre-chilled mortar and ground into a fine powder while maintaining the sample in a liquid nitrogen environment to prevent RNA degradation. The tissue powder was subsequently transferred to a centrifuge tube containing 1 mL of Trizol reagent. The tube was immediately vortexed for 30 s to ensure thorough mixing of the tissue and the reagent. After vortexing, the sample was allowed to stand at room temperature for 5 min. This incubation period facilitated the dissociation of the nucleic acid–protein complex. Next, 200 μL of chloroform (equivalent to 1/5 of the Trizol volume) was added to the tube. The tube was securely capped and shaken vigorously for 30–60 s until the mixture turned milky white, indicating proper emulsification. The sample was then centrifuged at 4 °C at a speed of 12,000× *g* for 15 min. After centrifugation, the sample stratified into three layers. The upper aqueous phase, which contained the RNA, was carefully aspirated (approximately 400–500 μL) and transferred to a new tube, taking care to avoid contact with the middle interphase layer. To precipitate the RNA, 0.5 mL of isopropanol (equal in volume to the Trizol used) was added to the tube containing the aqueous phase. The tube was gently inverted several times to ensure complete mixing, and then the sample was allowed to stand at room temperature for 10 min. Following this incubation, the sample was centrifuged again at 4 °C at 12,000× *g* for 10 min. A gel-like RNA precipitate was visible at the bottom of the tube after centrifugation. The supernatant was discarded, and the RNA precipitate was washed with 1 mL of 75% ethanol. The tube was then centrifuged at 4 °C at 7500× *g* for 5 min, and the supernatant was carefully removed. The tube was opened and inverted to air-dry the RNA precipitate at room temperature for 5–10 min. It was crucial to ensure that the precipitate did not dry completely, as it should remain translucent. Once the RNA precipitate was adequately dried, 20–30 μL of RNase-free water was added to dissolve the RNA. To assess the purity of the extracted total RNA, 1 μL of the RNA solution was loaded onto a detection plate of a NanoDrop spectrophotometer (Thermo Fisher Scientific, Waltham, MA, USA). The absorbance values at 260 nm and 280 nm were measured after adjusting the instrument to zero using DEPC-treated water. The purity of the total RNA was evaluated based on the A260/A280 ratio. A ratio between 1.8 and 2.0 indicated high-purity total RNA, while a ratio below 1.6 suggested contamination with proteins or organic phases.

### 2.5. RNA-Seq

#### 2.5.1. RNA Extraction and Sequencing for Expression Quantification

Total RNA integrity was assessed using an RNA Nano 6000 Assay Kit on the Bioanalyzer 2100 system (Agilent Technologies, Santa Clara, CA, USA). Sequencing libraries were prepared using total RNA as input material. Briefly, mRNA was enriched and purified via poly-T oligo-attached magnetic beads. The purified mRNA was fragmented using divalent cations under elevated temperature in First Strand Synthesis Reaction Buffer (5X) (New England BioLabs, Ipswich, MA, USA). First-strand cDNA was synthesized with random hexamer primers and M-MuLV Reverse Transcriptase (RNase H^−^), followed by second-strand cDNA synthesis using DNA Polymerase I and RNase H. The resulting cDNA fragments underwent end repair, adenylation at the 3′ ends, and ligation to Illumina adapters (Illumina, Inc., San Diego, CA, USA). cDNA fragments of ~370–420 bp in size were selected using the AMPure XP system (Beckman Coulter, Brea, CA, USA) and amplified by PCR with Phusion High-Fidelity DNA Polymerase, Universal PCR primers, and index primers. The final PCR products were purified, and library quality was verified using the Agilent Bioanalyzer 2100 system (Agilent Technologies, Santa Clara, CA, USA). Indexed libraries were clustered on a cBot Cluster Generation System (Illumina, Inc., San Diego, CA, USA) with a TruSeq PE Cluster Kit v3-cBot-HS (Illumina, Inc., San Diego, CA, USA) and sequenced on the Illumina Novaseq platform to generate 150 bp paired-end reads.

#### 2.5.2. RNA-Seq Data Analysis

The raw RNA-seq data in fastq format were first processed using fastp software 0.23.4 to perform quality control and filtering. This step generated clean reads by removing adapter sequences, poly-N reads, and low-quality reads. The quality of the cleaned data was assessed by calculating Q20, Q30 scores, and GC content. All subsequent analyses were based on these high-quality, clean data. Gene expression quantification was carried out by mapping the clean reads to the reference genome using featureCounts (v1.5.0-p3) [19] to count the number of reads mapped to each gene. Gene expression levels were estimated using the FPKM (Fragments Per Kilobase of transcript per Million mapped reads) method, which normalizes for both gene length and sequencing depth. Differential expression analysis between experimental conditions/groups was performed using the DESeq2 R package (v1.20.0), which employs a negative binomial distribution-based model to determine statistically significant differences in gene expression. The resulting *p*-values were adjusted for multiple testing using the Benjamini–Hochberg method to control the false discovery rate (FDR). Genes with an adjusted *p*-value ≤ 0.05 were considered differentially expressed. Functional enrichment analyses, including Gene Ontology (GO) terms and Kyoto Encyclopedia of Genes and Genomes (KEGG) pathways, were performed on the differentially expressed genes using the clusterProfiler [20] R package 4.3.3. The enrichment was evaluated based on the hypergeometric test. The resulting *p*-values were adjusted for multiple comparisons using the Benjamini–Hochberg method to control the false discovery rate (FDR). Terms or pathways with an adjusted *p*-value (FDR) < 0.05 were considered statistically significant.

### 2.6. Proteomics

#### 2.6.1. Protein Extraction

Brain tissue samples were pulverized in liquid nitrogen using a pre-cooled mortar and pestle. The powdered tissue was transferred to a chilled microcentrifuge tube containing SDT lysis buffer (4% SDS, 100 mM Tris-HCl, pH 7.6, 100 mM NaCl) and homogenized by brief vortexing. Subsequent lysis was performed via sonication in an ice-water bath for 5 min (3 s on, 7 s off cycles). The lysate was incubated at 95 °C for 8 min and then centrifuged at 12,000× *g* for 15 min at 4 °C. Iodoacetamide was added to the supernatant to a final concentration of 50 mM for alkylation, followed by incubation in the dark at room temperature for 1 h. Proteins were precipitated by adding four volumes of ice-cold acetone and incubating at −20 °C for at least 2 h. After centrifugation at 12,000× *g* for 15 min at 4 °C, the pellet was washed once with 1 mL of ice-cold acetone, air-dried briefly, and finally resuspended in Dissolved Buffer (DB buffer) for quantification.

#### 2.6.2. Proteomic Analysis

Tryptic peptides were separated using a nanoElute ultra-high-performance LC system (Bruker Daltonics, Billerica, MA, USA) coupled online to a timsTOF Pro2 mass spectrometer (Bruker Daltonics). The separation was performed on a C18-reversed-phase analytical column (25 cm length, 75 μm inner diameter, 1.9 μm resin) with the following gradient: 5–35% buffer B (99.9% acetonitrile and 0.1% formic acid) over 90 min, followed by a wash step increasing to 80% buffer B over 5 min. The flow rate was maintained at 300 nL/min throughout the analysis. The mass spectrometer was operated in PASEF (Parallel Accumulation-Serial Fragmentation) mode. MS1 spectra were collected over a mass range of *m*/*z* 100–1700, and up to 10 PASEF MS/MS scans were acquired per cycle in a data-dependent manner. Active exclusion was enabled with a release time of 24 s. Raw data were processed using MaxQuant [21] software (version 1.6.14.0) and searched against the Mus musculus UniProt proteome database (92,499 sequences). The search included label-free quantification (LFQ) and match between runs functions to maximize quantifiable proteins. The downstream analysis and visualization of the differentially expressed proteins (DEPs) were performed using customized scripts in R (version 3.4.3) and the DEP package (version 1.32.0).

### 2.7. Protein Sample Preparation and LC-MS/MS Analysis

The digested peptide samples were analyzed using a nanoElute ultra-high-performance liquid chromatography (UHPLC) system (Bruker Daltonics) coupled online to a timsTOF Pro 2 mass spectrometer (Bruker Daltonics). Peptides were separated on a reversed-phase C18 column with a 90 min linear gradient. The mass spectrometer was operated in parallel accumulation–serial fragmentation (PASEF) mode. Full MS scans were acquired over a mass range of *m*/*z* 100–1700. For MS/MS acquisition, the top 10 most intense precursors per cycle were selected for fragmentation, with a total cycle time of 1.17 s. Other key instrument parameters included the following: ramp time, 100 ms; lock duty cycle, 100%; ion intensity threshold for MS/MS, 2500; and scheduling target intensity, 20,000. Raw mass spectrometry data were generated in the end.

## 3. Results

### 3.1. The Flowchart of This Study

**Figure 1:**
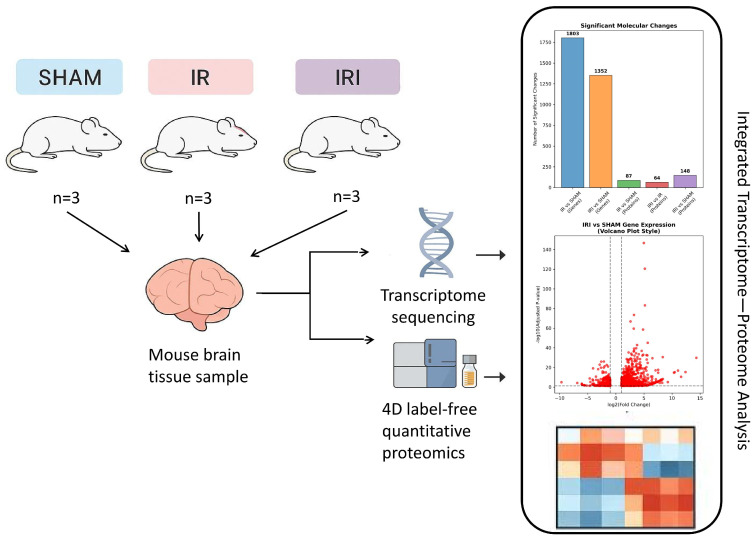
A graphical overview of the experimental timeline and analytical procedures. SHAM: SHAM-operated controls; IR: permanent middle cerebral artery occlusion; IRI: transient occlusion with reperfusion.

A graphical overview of the experimental timeline and analytical procedures is provided in Figure 1. This study was designed to compare molecular profiles across three experimental groups: SHAM-operated controls (SHAM), permanent middle cerebral artery occlusion (IR), and transient occlusion with reperfusion (IRI). Following the establishment and validation of the model, cortical tissue samples were carefully collected from the ischemic region to undergo RNA Sequencing and Proteomics Sequencing. Additionally, the sequencing data were subjected to in-depth bioinformatic analyses, integrating transcriptomic and proteomic approaches to identify differentially expressed genes and proteins, as well as their potential functional implications. The downstream data processing and integrative bioinformatics pipeline are depicted in the flowchart.

### 3.2. Validation of Successful MCAO Model Induction Through Cerebral Blood Flow Reduction

**Figure 2:**
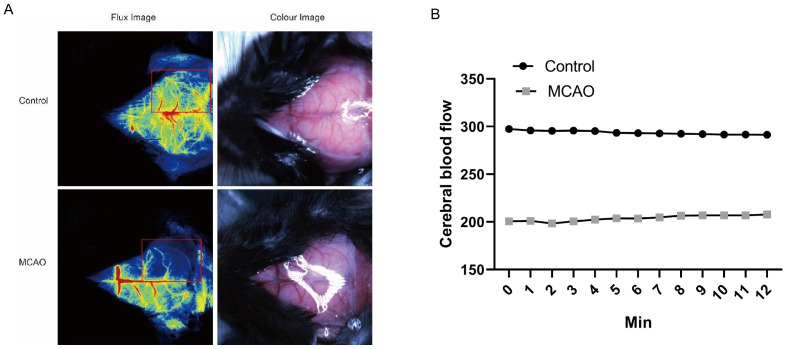
Intraoperative monitoring of cerebral blood flow confirms successful MCAO (middle cerebral artery occlusion) model induction. (**A**) The blood flow images before and after modeling were monitored by a laser speckle rheometer, and the blood flow in the MCAO group was significantly lower than that in the control group. (**B**) The cerebral blood flow in the MCA (middle cerebral artery) area was monitored by laser Doppler flowmetry, and the blood flow in the MCAO group was significantly lower than that in the control group.

The Laser Doppler Bloody Oxygen System–Laser Speckle Blood Flow Imaging System is used to monitor the local blood flow of MCA in real time during the operation and continuously monitor the changes in cerebral blood flow in the infarcted area during the whole process of middle cerebral artery occlusion (MCAO) operation. The decrease in cerebral blood flow in the MCAO area can be found in speckle images of animals that have successfully established MCAO models. At the same time, the monitoring of cerebral blood flow in the MCA area by laser Doppler flowmetry showed that the cerebral blood flow in the MCA area also decreased significantly (Figure 2A,B). This evidence strongly supports the successful induction of cerebral ischemia in the MCAO model animals.

### 3.3. Transcriptomic Analysis of the Three Groups (SHAM, IR, and IRI)

**Figure 3:**
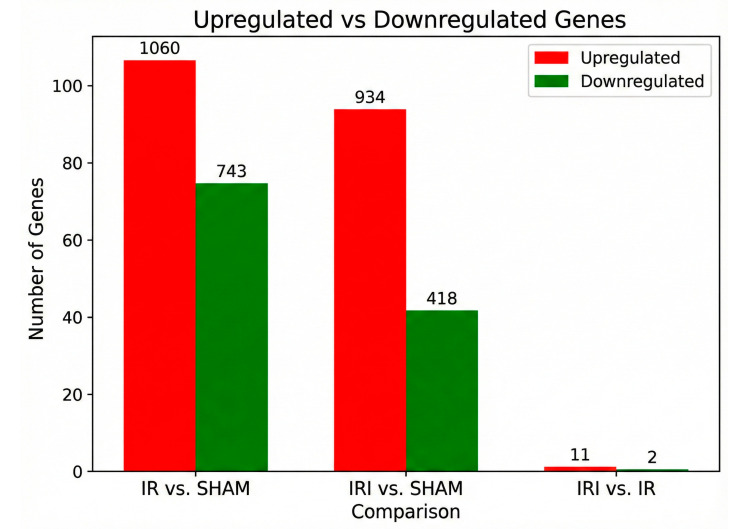
Summary of differential gene expression across experimental conditions. A bar chart illustrating the number of differentially expressed genes (DEGs) identified in each comparison. The IR group (permanent ischemia) versus SHAM (SHAM-operated controls) shows the largest number of alterations (1803 DEGs). The IRI group (ischemia–reperfusion) versus SHAM shows a substantial but reduced number of changes (1352 DEGs). The direct comparison of IRI vs. IR, isolating the reperfusion-specific effect, reveals a focused set of 13 DEGs. Within each bar, upregulated genes are shown in red and downregulated genes in green.

The mouse model was divided into SHAM, IR, and IRI groups and subjected to corresponding treatments. We observed that the most critical transcriptomic changes occurred following 24 h of permanent ischemia (IR) and ischemia–reperfusion injury (IRI), as determined by assessing differential gene expression using established thresholds. We used FDR of <0.05 and |FC| of >2.0 as thresholds for assigning significant differences. The results showed that cerebral tissue exhibited different transcriptomic characteristics during ischemia and ischemia–reperfusion. Compared to the SHAM group, the IR group had 1803 DEGs, with 1060 upregulated genes and 743 downregulated genes. Compared to the SHAM group, the IRI group had 1352 DEGs, with 934 upregulated and 418 downregulated genes. Most notably, the reperfusion-specific comparison (IRI vs. IR) revealed 13 DEGs, with 11 upregulated and 2 downregulated genes (Figure 3; Supplementary Tables S2–S10). A Venn diagram showing gene overlap between comparisons, volcano plots for each contrast, and pathway enrichment analyses performed separately for common and condition-specific DEGs are provided in Supplementary Figures S1–S5.

To quantitatively evaluate the inflammatory response, we directly compared the expression fold-changes of key immune mediators between IR and IRI conditions (vs. SHAM). While hallmark inflammatory genes, such as Saa3 (IR: 13.95-fold, IRI: 14.33-fold), Lcn2 (IR: 11.55-fold, IRI: 11.61-fold), and Cxcl10, were robustly upregulated in both injury states, a subset of acute phase reactants exhibited consistently greater induction in IRI. For example, Marco was upregulated 11.48-fold in IR but 12.40-fold in IRI. This quantitative analysis confirms a shared core inflammatory program while revealing that reperfusion selectively amplifies specific components, particularly those involved in innate immune recognition and acute phase signaling.

**Figure 4:**
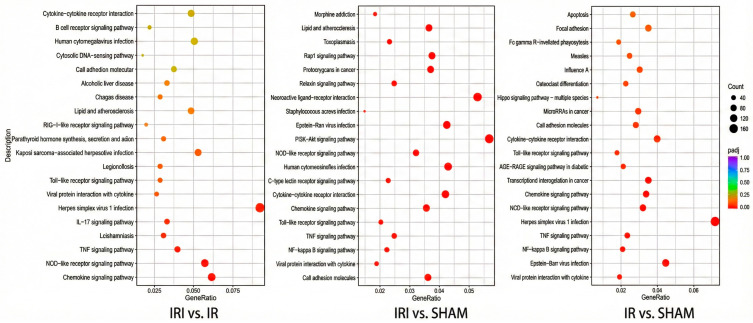
KEGG analysis of DEGs among the three transcriptomic groups.

KEGG pathway enrichment analysis based on differentially expressed genes (DEGs) revealed distinct pathway activation patterns across the three experimental comparisons (Q value ≤ 0.05). In the IR versus SHAM comparison, the most significantly enriched pathways included the TNF signaling pathway (Q-value: 1.36 × 10^−4^), viral protein interaction with cytokine and cytokine receptor (Q-value: 1.36 × 10^−4^), and NF-kappa B signaling pathway (Q-value: 2.65 × 10^−2^) (Supplementary Table S11). The IRI versus SHAM comparison demonstrated even more pronounced inflammatory pathway enrichment, with the top pathways being the TNF signaling pathway (Q-value: 5.49 × 10^−5^), viral protein interaction with cytokine and cytokine receptor (Q-value: 1.09 × 10^−3^), and the NF-kappa B signaling pathway (Q-value: 2.26 × 10^−3^) (Supplementary Table S12). Notably, the IRI versus IR comparison, despite having fewer DEGs, revealed enrichment in distinct biological processes. The most significant pathways included human T-cell leukemia virus 1 infection (Q-value: 5.21 × 10^−2^), glycolysis/gluconeogenesis (Q-value: 7.28 × 10^−2^), and amphetamine addiction (Q-value: 7.28 × 10^−2^). Additional relevant pathways encompassed RNA degradation, the GnRH signaling pathway, and the IL-17 signaling pathway (all Q-value: 7.28 × 10^−2^) (Supplementary Table S13). The consistent enrichment in TNF signaling and the NF-κB pathways across multiple comparisons highlights the central role of inflammatory responses in cerebral reperfusion injury (Figure 4). Furthermore, the unique pathway profile in the IRI versus IR comparison suggests the involvement of metabolic reprogramming and neuronal signaling mechanisms during the progression from ischemia to reperfusion injury.

**Figure 5:**
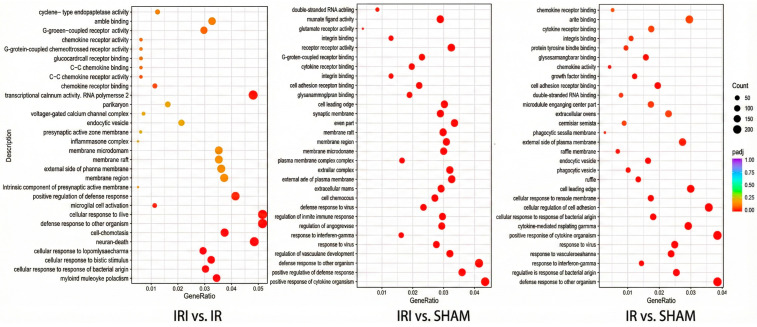
GO (Gene Ontology) analysis of DEGs (differentially expressed genes) among the three transcriptomic groups. The dot plot integrates the enrichment results from all three ontology domains: biological process (BP), cellular component (CC), and molecular function (MF). Each dot represents a significantly enriched GO term.

GO enrichment analysis of differentially expressed genes across the three comparative groups revealed both shared and distinct biological themes. In the IRI versus IR comparison (Supplementary Table S14), the most significantly enriched biological processes included myeloid leukocyte activation, cellular response to lipopolysaccharide, and neuron death. Cellular components were predominantly associated with presynaptic membrane structures, inflammasome complexes, and membrane microdomains, while molecular functions highlighted chemokine receptor binding and G protein-coupled receptor activity. The IRI versus SHAM comparison (Supplementary Table S15) showed enrichment in biological processes related to positive regulation of cytokine production, defense response to virus, and regulation of the innate immune response. Cellular components were characterized by the extracellular matrix, synaptic membranes, and plasma membrane receptor complexes, with molecular functions involving cytokine receptor binding, integrin binding, and glutamate receptor activity. Similarly, the IR versus SHAM comparison (Supplementary Table S16) demonstrated significant enrichment in defense response to other organisms, response to interferon-gamma, and the cytokine-mediated signaling pathway. Cellular components featured cell leading edge structures, phagocytic vesicles, and external plasma membrane regions, while molecular functions emphasized chemokine activity, chemokine receptor binding, and cytokine receptor binding. These multi-group GO results collectively demonstrate that neuro-inflammatory responses represent a fundamental mechanism underlying cerebral reperfusion injury at the transcriptomic level. The consistent enrichment in immune and inflammatory processes across all comparisons, coupled with the specific involvement of neuronal signaling pathways and synaptic structures in the IRI versus IR group, highlights the complex interplay between neuroinflammation and neuronal dysfunction during the progression from ischemia to reperfusion injury (Figure 5).

### 3.4. Proteomic Analysis of the Three Groups (SHAM, IR, and IRI)

By using a threshold of 1.5-fold change and a *p*-value of <0.05, comprehensive proteomic profiling across the three experimental groups revealed distinct molecular signatures associated with cerebral ischemia and reperfusion injury. A comparison between the IRI and SHAM groups identified the most extensive proteomic alterations, with 324 differentially expressed proteins (DEPs), highlighting robust activation of the acute phase response, complement system, and interferon signaling pathways. Key upregulated proteins included serum amyloid A-1 (FC = 32.07), haptoglobin (FC = 12.85), and interferon-induced protein with tetratricopeptide repeats 3 (FC = 10.49), indicating massive inflammatory activation and immune response during reperfusion injury (Supplementary Table S5).

In contrast, the IR versus SHAM comparison showed a moderate response with 192 DEPs, characterized by pronounced interferon signaling activation but minimal acute phase response, as evidenced by the upregulation of interferon-induced transmembrane protein 3 (FC = 7.71) and vesicular glutamate transporter 2 (FC = 7.45). This pattern suggests that permanent ischemia primarily triggers interferon-mediated immune mechanisms without the extensive acute phase response observed in reperfusion injury (Supplementary Table S2). The transition from permanent ischemia to reperfusion (IRI vs. IR) involved more specific molecular changes, with only 142 DEPs identified. These changes included the upregulation of adhesion G protein-coupled receptor L2 (FC = 7.99) and coiled–coil–helix–coiled–coil–helix domain-containing protein 1 (FC = 7.12), indicating activation of cellular adhesion mechanisms and early vascular repair processes during reperfusion (Supplementary Table S3).

**Figure 6:**
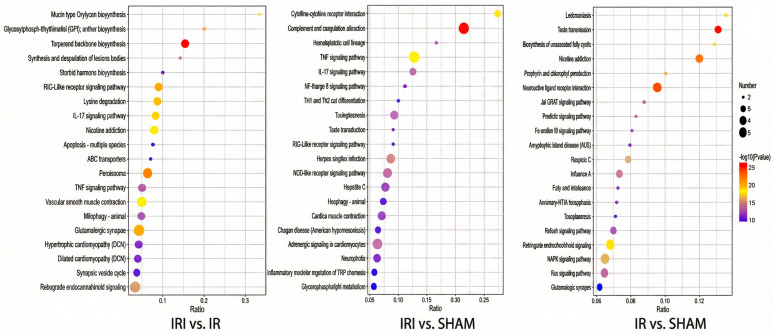
KEGG analysis of DEGs among the three proteomics groups.

Collectively, these proteomic findings demonstrate that cerebral ischemia–reperfusion injury triggers a massive inflammatory response characterized by acute phase protein activation and complement system engagement, while permanent ischemia induces a more focused interferon-mediated immune response. The specific proteomic changes observed during the transition from ischemia to reperfusion highlight the activation of vascular repair and cellular adhesion mechanisms, providing insights into the molecular events underlying reperfusion injury progression (Figure 6).

### 3.5. Integrative Analysis of the Transcriptomics and Proteomics Data

**Figure 7:**
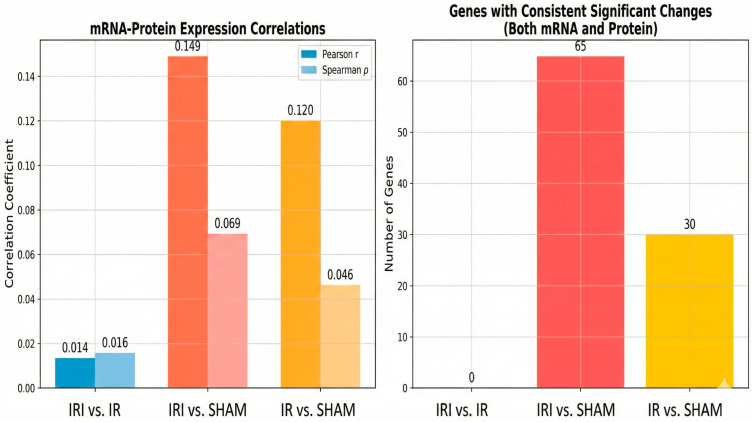
Scatter plots depicting the correlation between mRNA expression (*X*-axis) and protein expression (*Y*-axis) levels for IRI vs. IR, IRI vs. SHAM, and IR vs. SHAM comparisons. SHAM: SHAM-operated controls, IR: permanent middle cerebral artery occlusion, and IRI: transient occlusion with reperfusion.

**Figure 8:**
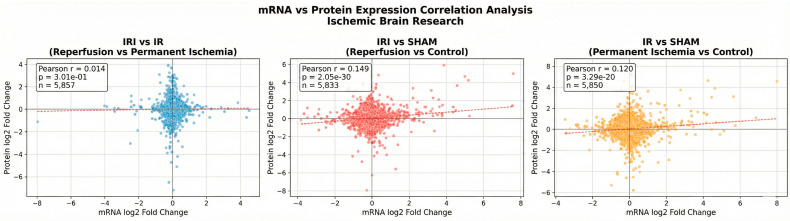
Scatter plots illustrating the correlation between mRNA expression (*X*-axis) and protein expression (*Y*-axis) levels for the same comparisons as in Figure 7, analyzed using Pearson correlation. SHAM: SHAM-operated controls, IR: permanent middle cerebral artery occlusion, and IRI: transient occlusion with reperfusion.

To investigate correlations between mRNA expression and protein expression levels, we performed a combined transcriptomic and proteomic analysis of the data from three experimental comparisons: IRI vs. IR (reperfusion vs. permanent ischemia), IRI vs. SHAM (reperfusion vs. control), and IR vs. SHAM (permanent ischemia vs. control). We generated scatter plots of the mRNA expression levels of known genes (*X*-axis) versus the corresponding protein expression levels (*Y*-axis) to compare protein versus mRNA abundances across all three comparisons. The correlation values calculated using Spearman’s rank correlation coefficient test revealed varying degrees of correlation: IRI vs. IR showed weak correlation (ρ = 0.016, *p* = 0.224), while IRI vs. SHAM (ρ = 0.069, *p* = 1.18 × 10^−7^) and IR vs. SHAM (ρ = 0.046, *p* = 3.82 × 10^−4^) demonstrated statistically significant but modest correlations (Figure 7). Pearson correlation analysis yielded similar patterns, with IRI vs. SHAM showing the strongest correlation (r = 0.149, *p* = 2.05 × 10^−30^), followed by IR vs. SHAM (r = 0.120, *p* = 3.29 × 10^−20^) and IRI vs. IR (r = 0.014, *p* = 0.301) (Figure 8).

Integrative analysis identified 65 genes in the IRI vs. SHAM comparison and 30 genes in the IR vs. SHAM comparison that were differentially expressed at both the mRNA and protein levels with consistent directionality (|log2FC| > 0.5, *p* < 0.05). Notably, 16 genes showed consistent significant changes across multiple comparisons, including Cp, Galnt16, Ifit1, Ifit2, Ifit3, Irgm1, Mt2, and Saa3. Among the IRI vs. SHAM comparison, the most prominent concordantly upregulated genes included Saa3, Lcn2, Saa1, Serpina3n, and Hp (Supplementary Tables S17–S19). These molecules are primarily involved in acute phase response, inflammatory signaling, interferon response, and oxidative stress pathways, highlighting their crucial roles in cerebral ischemia–reperfusion injury. The modest correlations between mRNA and protein levels across most genes suggest complex post-transcriptional regulatory mechanisms during cerebral ischemia–reperfusion, including potential translational control, protein stability differences, post-translational modifications, and temporal delays between transcription and translation that may influence the final protein abundance independent of transcript levels.

The genes showing concordant regulation at both the transcriptional and translational levels represent key mediators of cerebral ischemia and reperfusion injury. Saa3 (Serum amyloid A3) showed the most dramatic upregulation (14.33-fold at the mRNA level, with protein levels reaching detection limits in IRI vs. SHAM), indicating its potential as a central inflammatory mediator in stroke pathology. Lcn2 (Lipocalin-2) also demonstrated substantial coordinated upregulation (11.61-fold at mRNA, protein reaching detection limits), suggesting its critical involvement in neuroinflammation and iron metabolism dysregulation. Saa1 showed robust elevation at both levels (7.58-fold mRNA, 5.00-fold protein), further supporting the activation of acute phase response pathways. Interferon-induced genes (Ifit1, Ifit2, Ifit3) demonstrated consistent upregulation across multiple comparisons, indicating sustained antiviral and inflammatory responses (Supplementary Table S20). The metallothionein Mt2 and ceruloplasmin (Cp) showed concordant changes, supporting the activation of oxidative stress response and metal ion homeostasis pathways during ischemic injury. These concordantly regulated molecules represent the most robust biomarkers of cerebral ischemia–reperfusion injury and potential therapeutic targets for intervention, as their coordinated regulation at both the transcriptional and translational levels indicates fundamental biological responses to ischemic insult.

## 4. Discussion

### 4.1. Molecular Landscape of Cerebral Ischemia and Reperfusion

Our comprehensive multi-omics analysis reveals the intricate molecular complexity underlying cerebral ischemia and reperfusion injury, challenging traditional conceptualizations of ischemic brain damage. This study’s unique experimental design, comparing SHAM control, permanent ischemia (IR), and ischemia–reperfusion (IRI) conditions, provides unprecedented insights into the molecular mechanisms of cerebrovascular injury. While our findings align with prior studies in highlighting inflammation as a central mechanism—particularly the TNF signaling pathway and cytokine–cytokine receptor interaction, as reported in endothelial-focused research [22,23]—we further uncover reperfusion-specific molecular signatures that distinguish it from permanent ischemia [24,25,26]. The limited transcriptomic changes (13 DEGs) but substantial proteomic alterations (142 DEPs) in IRI versus IR underscore the dominance of post-transcriptional regulation during reperfusion, a phenomenon that may reflect the more complex in vivo environment compared to cell-based models [27,28].

### 4.2. Transcriptional and Proteomic Dynamics

Transcriptomic analysis confirmed a substantial shared inflammatory trigger in both IR and IRI, with key mediators like Saa3 and Lcn2 highly upregulated. However, our quantitative comparison and proteomic data reveal critical distinctions. First, reperfusion (IRI) uniquely amplifies a specific acute phase component (e.g., higher Marco induction). Second, and more decisively, the proteome shows a qualitative switch: IR is defined by an interferon signature, while IRI is defined by an acute phase/complement storm. This shift suggests reperfusion injury transitions the tissue toward a more destructive, sterile inflammatory state. Furthermore, IRI specifically upregulated neuroprotective genes (Arc, Npas4) and showed profound metabolic reprogramming (Gapdh downregulation), programs largely absent in the IR transcriptome. Most strikingly, the extreme transcriptome–proteome decoupling (r = 0.014 in IRI vs. IR) underscores post-transcriptional regulation as a hallmark of the reperfusion phase, a regulatory layer not predominant in permanent ischemia. Proteomic profiling revealed a fundamental qualitative shift in the dominant inflammatory signature. The IRI state was characterized by a massive acute phase and complement system eruption (e.g., Serum Amyloid A-1, SAA1, FC = 32.07; Haptoglobin, FC = 12.85). In stark contrast, the IR proteome was dominated by a potent interferon-stimulated gene (ISG) signature (e.g., IFITM3, FC = 7.71; IFIT3, FC = 10.49) with minimal acute phase protein involvement. This indicates that while both conditions activate immune pathways, reperfusion injury pivots the response toward a more tissue-disruptive, systemic inflammatory cascade, whereas permanent ischemia sustains a targeted, antiviral-like interferon response. Notably, while both our study and previous endothelial research [22] identified ECM disruption as a key feature, our data revealed distinct patterns: rather than the THBS1 upregulation observed in endothelial models, we detected significant MMP activation (Mmp3, Mmp12) and broader tissue remodeling pathways. Similarly, complement system activation emerged as a shared mechanism, though our transcriptomic data (C3, C4b upregulation) complemented the proteomic evidence from earlier studies. The consistent observation of poor mRNA–protein correlation across studies (r = 0.492 in Ji et al. [22] versus similar discordance in our data) reinforces the importance of post-transcriptional regulation in reperfusion injury.

### 4.3. Reperfusion: A Unique Molecular Phenomenon

The identification of reperfusion-specific molecular changes challenges the notion of reperfusion as a mere reversal of ischemic damage [29,30]. The upregulation of neuroprotective genes, such as Pisd-ps3 (2.10-fold), Arc (1.47-fold), and Npas4 (2.09-fold), indicates an adaptive molecular response aimed at mitigating secondary injury [31,32]. Notably, the significant proteomic changes (142 DEPs) in IRI versus IR, including the upregulation of adhesion G protein-coupled receptor L2 (FC = 7.99) and CHCHD1 (FC = 7.12), suggest activation of vascular repair mechanisms [33,34,35]. This reperfusion-specific molecular profile, involving both neuroprotection and vascular remodeling, diverges from the endothelial-centric mechanisms reported in earlier studies and underscores the multifaceted nature of reperfusion injury [36]. Perhaps our most significant advancement lies in characterizing reperfusion as a distinct molecular phenomenon rather than merely an extension of ischemic damage. The modest transcriptional changes (13 DEGs in IRI vs. IR) contrast sharply with the 390 DEGs reported in endothelial cell models [22], potentially reflecting the integrated tissue response in our in vivo system. More importantly, we identified a unique repertoire of neuroprotective genes (Arc, Npas4) specifically upregulated during reperfusion, suggesting activation of endogenous repair mechanisms concurrent with injury processes [37]. This dual nature of reperfusion—simultaneously destructive and reparative—represents a conceptual advance beyond the predominantly injury-focused perspective of previous research.

### 4.4. Pathway Analysis and Mechanistic Insights

Our pathway analysis not only confirmed established inflammatory mechanisms but also revealed novel aspects of reperfusion biology. While prior research emphasized TGF-β and JAK-STAT signaling [22,38], we identified dramatic enrichment in type I interferon pathways (*p* = 1.10 × 10^−15^) and viral defense responses (*p* = 2.94 × 10^−16^), suggesting reperfusion injury may mimic antiviral immune activation [39,40,41]. Additionally, the prominent metabolic reprogramming observed in our data, particularly Gapdh downregulation (4.03-fold), indicating glycolytic suppression, represents a mechanism largely unexplored in endothelial-focused studies [42,43]. Under the extreme stress of reperfusion, cells may actively shut down normal energy metabolism and divert resources and metabolic flux to other more urgent survival programs. This could represent an adaptive self-protection mechanism or, alternatively, may reflect direct “paralysis” of the metabolic machinery due to damage from oxidative stress. Given that normal endothelial cell function is highly energy-dependent, this abrupt halt in metabolic activity inevitably severely compromises vascular integrity, barrier function, and signaling capacity, thereby exacerbating injury. This reperfusion-specific metabolic paralysis, exemplified by Gapdh downregulation, may not only compromise acute cellular survival but also contribute to sustained cerebrovascular dysfunction. Impaired endothelial energy metabolism is a known precursor to vascular remodeling and stiffness, which are clinically measurable as increased carotid intima-media thickness—a marker linked to reduced cerebral oxygenation in humans [44]. Thus, our identified metabolic reprogramming may represent a molecular bridge between acute IRI and long-term cerebrovascular damage. While previous research has predominantly focused on inflammation, our work addresses this gap by revealing the fundamental role of this metabolic adaptation. These findings collectively support a revised mechanistic model encompassing three core processes:

(1) Inflammatory Cascade Activation: The massive upregulation of cytokines and chemokines, coupled with interferon response pathway activation, indicates a complex inflammatory response extending beyond traditional signaling [45]. The IRI-specific upregulation of Saa3 and Lcn2 highlights their roles as central mediators of reperfusion-associated inflammation [46,47].
(2) Tissue Remodeling Processes: Matrix metalloproteinase activation (e.g., Mmp3, FC = 11.24 in IR/SHAM) and extracellular matrix modifications suggest active tissue repair concurrent with inflammatory responses [48,49]. These processes align with Ji et al.’s findings on ECM disruption but are uniquely amplified in reperfusion [22].
(3) Metabolic Reprogramming: The downregulation of Gapdh (4.03-fold) and enrichment in glycolysis/gluconeogenesis pathways in IRI versus IR highlight metabolic adaptations during reperfusion [50], a mechanism less emphasized in prior endothelial-focused studies.

Notably, these reperfusion-specific molecular disturbances—particularly the metabolic shutdown and intense sterile inflammation—may directly underlie the cerebrovascular dysfunction observed in clinical settings. Studies have shown that impaired cerebral oxygenation during physiological stress is associated with early markers of systemic vascular injury, such as increased carotid intima-media thickness and arterial stiffness [44]. Our data suggest that the reperfusion-induced molecular storm we describe could be a key mechanistic driver of such subclinical vascular damage, thereby bridging acute molecular pathology with the progression of long-term cerebrovascular disease.

### 4.5. Therapeutic Implications and Precision Medicine

This study unveils promising therapeutic targets, including Saa3, Marco, Lcn2, Pisd-ps3, and Npas4. The distinct molecular profiles between ischemia and reperfusion suggest the potential for timing-specific therapeutic approaches [51,52]. For example, targeting TNF-α (as demonstrated by adalimumab in Ji et al. [22]) may mitigate reperfusion-induced endothelial inflammation, while interventions against Saa3 or interferon pathways could address reperfusion-specific injury. The strong interferon signature and metabolic shifts also open avenues for the following:

(1) Drug Repurposing: Anti-inflammatory agents (e.g., JAK inhibitors) and metabolic modulators.
(2) Biomarker Development: Saa3 and Lcn2 as biomarkers for injury progression.
(3) Combination Therapies: Targeting both inflammatory and neuroprotective pathways.

### 4.6. Limitations and Future Directions

While this study provides unprecedented molecular insights, limitations inherent to mouse model research remain. Future investigations should validate these findings in human tissues, develop targeted interventions against reperfusion-specific molecules like Saa3 and Pisd-ps3, and functionally explore the roles of neuroprotective genes (Arc, Npas4) and metabolic reprogramming in reperfusion recovery and neuronal repair.

## 5. Conclusions

This study redefines cerebral ischemia–reperfusion injury by revealing its molecular heterogeneity and reperfusion-specific mechanisms. Beyond the acute phase, the reperfusion-specific molecular landscape we describe—marked by severe inflammatory and metabolic shifts—may serve as a critical link between acute ischemic insults and long-term cerebrovascular dysfunction. This aligns with clinical studies associating cerebral oxygenation deficits with markers of preclinical vascular injury, such as arterial stiffness and retinal microvascular alterations [44]. By integrating transcriptomic and proteomic data, we provide a robust foundation for developing precision therapeutics that bridge neuroprotection, immunomodulation, and metabolic regulation. The proposed mechanistic model encapsulates these insights, offering a roadmap for future research and clinical innovation aimed at mitigating both acute reperfusion injury and its potential long-term vascular consequences.

## Data Availability

The original contributions presented in this study are included in the article/Supplementary Materials. Further inquiries can be directed to the corresponding authors.

## Supplementary Materials

The following supporting information can be downloaded at https://www.mdpi.com/article/10.3390/cimb48010124/s1.

## Abbreviations

The following abbreviations are used in this manuscript:

IRI	Cerebral ischemia–reperfusion injury
IR	Cerebral permanent ischemia
SHAM	Surgically handled and manipulated controls
MCAO	Middle cerebral artery occlusion
DEPs	Differentially expressed proteins
DEGs	Differentially expressed genes

## References

[1] She R., Liu D., Liao J., Wang G., Ge J., Mei Z. (2023). Mitochondrial dysfunctions induce PANoptosis and ferroptosis in cerebral ischemia/reperfusion injury: From pathology to therapeutic potential. Front. Cell. Neurosci..

[2] Xu H., Lu X., Qin R., Shao L., Chen L. (2025). The Evolution of Ischemia-Reperfusion Injury Research in Ischemic Stroke: Insights from a Two-Decade Bibliometric analysis. Brain Behav..

[3] Meng X., Zheng Z., Yang L., Yang C., Li X., Hao Y. (2025). Dynamic mechanisms and targeted interventions in cerebral ischemia–reperfusion injury: Pathological cascade from ischemia to reperfusion and promising therapeutic strategies. Front. Neurosci..

[4] Shaik N.F., Regan R.F., Naik U.P. (2021). Platelets as drivers of ischemia/reperfusion injury after stroke. Blood Adv..

[5] Jiang Z., Chen Q., Yang H. (2025). Drug delivery strategies for neuroprotective therapy in ischemic stroke: Application of nanotechnology. Neural Regen. Res..

[6] Li J., Wei G., Song Z., Chen Z., Gu J., Zhang L., Wang Z. (2024). SIRT5 Regulates Ferroptosis through the Nrf2/HO-1 Signaling Axis to Participate in Ischemia-Reperfusion Injury in Ischemic Stroke. Neurochem. Res..

[7] Si W., You R., Sun B., Luo J., Hu G. (2024). The role of LCN2 in exacerbating ferroptosis levels in acute ischemic stroke injury. Biochem. Biophys. Res. Commun..

[8] Han B., Zhou S., Zhang Y., Chen S., Xi W., Liu C., Zhou X., Yuan M., Yu X., Li L. (2024). Integrating spatial and single-cell transcriptomics to characterize the molecular and cellular architecture of the ischemic mouse brain. Sci. Transl. Med..

[9] Yao X., Feng S.-Q., Fan B.-Y., Pang Y.-L., Li W.-X., Zhao C.-X., Zhang Y., Wang X., Ning G.-Z., Kong X.-H. (2020). Liproxstatin-1 is an effective inhibitor of oligodendrocyte ferroptosis induced by inhibition of glutathione peroxidase 4. Neural Regen. Res..

[10] Fu C., Wu Y., Liu S., Luo C., Lu Y., Liu M., Wang L., Zhang Y., Liu X. (2022). Rehmannioside A improves cognitive impairment and alleviates ferroptosis via activating PI3K/AKT/Nrf2 and SLC7A11/GPX4 signaling pathway after ischemia. J. Ethnopharmacol..

[11] Gao N., Huang Z., Xie J., Gao S., Wang B., Feng H., Bao C., Tian H., Liu X. (2025). Cryptotanshinone alleviates cerebral ischemia reperfusion injury by regulating ferroptosis through the PI3K/AKT/Nrf2 and SLC7A11/GPX4 signaling pathway. J. Ethnopharmacol..

[12] Yu Y., Xie X., Zhang Y., Xiao S., Dan F., Liu J., Liu Z., Zhou R. (2025). Salvigenin mitigates neuronal ferroptosis by binding to PI3K and enhancing the interaction between VCP and PI3K in the repair of spinal cord injury. Phytomedicine.

[13] Liu S.-Z., He X.-M., Zhang X., Zeng F.-C., Wang F., Zhou X.-Y. (2016). Ischemic Preconditioning-Induced SOCS-1 protects rat intestinal ischemia reperfusion injury via degradation of TRAF6. Dig. Dis. Sci..

[14] Zhao Q., Cui Z., Zheng Y., Li Q., Xu C., Sheng X., Tao M., Xu H. (2017). Fenofibrate protects against acute myocardial I/R injury in rat by suppressing mitochondrial apoptosis as decreasing cleaved caspase-9 activation. Cancer Biomark..

[15] Weng Z., Wang Y., Ouchi T., Liu H., Qiao X., Wu C., Zhao Z., Li L., Li B. (2022). Mesenchymal Stem/Stromal cell senescence: Hallmarks, mechanisms, and combating strategies. Stem Cells Transl. Med..

[16] Zhang Y., Judeh R., Aravind S., Zreiqat H., Lu Z. (2025). Mesenchymal stem cell senescence and Biomaterial-Based Next-Generation rejuvenation Strategy. Tissue Eng. Part B Rev..

[17] Sugimoto K., Chung D.Y., Fischer P., Takizawa T., Qin T., Yaseen M.A., Sakadžić S., Ayata C. (2024). Optogenetic Functional Activation Is Detrimental during Acute Ischemic Stroke in Mice. Stroke.

[18] Zhang X., Pei J., Xue L., Zhao Z., Xu R., Zhang C., Zhang C., Fu L., Zhang X., Cui L. (2024). An-Gong-Niu-Huang-Wan (AGNHW) Regulates Cerebral Blood Flow by Improving Hypoperfusion, Cerebrovascular Reactivity and Microcirculation Disturbances after Stroke. Chin. Med..

[19] Liao Y., Smyth G.K., Shi W. (2014). featureCounts: An Efficient General Purpose Program for Assigning Sequence Reads to Genomic Features. Bioinformatics.

[20] Yu G., Wang L.-G., Han Y., He Q.-Y. (2012). clusterProfiler: An R Package for Comparing Biological Themes among Gene Clusters. OMICS.

[21] Tyanova S., Temu T., Cox J. (2016). The MaxQuant Computational Platform for Mass Spectrometry-Based Shotgun Proteomics. Nat. Protoc..

[22] Ji Y., Chen Y., Tan X., Huang X., Gao Q., Ma Y., Yang S., Yin M., Yu M., Fang C. (2024). Integrated Transcriptomic and Proteomic Profiling Reveals the Key Molecular Signatures of Brain Endothelial Reperfusion Injury. CNS Neurosci. Ther..

[23] Wang L., Chen Y., Liu Y., Wei J., Miao L., Chang Z., Jia M., Liu L., Liang X., Zhang Y. (2025). Traditional Chinese Medicine for Cerebral Ischemia-Reperfusion Injury Prevention: Molecular Mechanisms and Future Perspectives. Am. J. Chin. Med..

[24] Tian X., Yang W., Jiang W., Zhang Z., Liu J., Tu H. (2024). Multi-Omics Profiling Identifies Microglial Annexin A2 as a Key Mediator of NF-κB pro-Inflammatory Signaling in Ischemic Reperfusion Injury. Mol. Cell. Proteom..

[25] Dai F., Hu C., Li X., Zhang Z., Wang H., Zhou W., Wang J., Geng Q., Dong Y., Tang C. (2024). Cav3.2 Channel Regulates Cerebral Ischemia/Reperfusion Injury: A Promising Target for Intervention. Neural Regen. Res..

[26] Kelmanson I.V., Shokhina A.G., Kotova D.A., Pochechuev M.S., Ivanova A.D., Kostyuk A.I., Panova A.S., Borodinova A.A., Solotenkov M.A., Stepanov E.A. (2021). In Vivo Dynamics of Acidosis and Oxidative Stress in the Acute Phase of an Ischemic Stroke in a Rodent Model. Redox Biol..

[27] Xie W., Zhu T., Zhou P., Xu H., Meng X., Ding T., Nan F., Sun G., Sun X. (2023). Notoginseng Leaf Triterpenes Ameliorates Mitochondrial Oxidative Injury via the NAMPT-SIRT1/2/3 Signaling Pathways in Cerebral Ischemic Model Rats. J. Ginseng Res..

[28] Li T.-H., Sun H.-W., Song L.-J., Yang B., Zhang P., Yan D.-M., Liu X.-Z., Luo Y.-R. (2022). Long Non-Coding RNA MEG3 Regulates Autophagy after Cerebral Ischemia/Reperfusion Injury. Neural Regen. Res..

[29] Lei Y., Zhang X., Ni W., Gao C., Li Y., Yang H., Gao X., Xia D., Zhang X., Osipowicz K. (2024). Application of Individual Brain Connectome in Chronic Ischemia: Mapping Symptoms before and after Reperfusion. MedComm.

[30] Polonsky M., Gerhardt L.M.S., Yun J., Koppitch K., Colón K.L., Amrhein H., Wold B., Zheng S., Yuan G.-C., Thomson M. (2024). Spatial Transcriptomics Defines Injury Specific Microenvironments and Cellular Interactions in Kidney Regeneration and Disease. Nat. Commun..

[31] Fouda A.Y., Xu Z., Suwanpradid J., Rojas M., Shosha E., Lemtalsi T., Patel C., Xing J., Zaidi S.A., Zhi W. (2022). Targeting Proliferative Retinopathy: Arginase 1 Limits Vitreoretinal Neovascularization and Promotes Angiogenic Repair. Cell Death Dis..

[32] Wang D., Zhao Q., Qin J., Guo Y., Zhang C., Li Y. (2022). Urokinase Loaded Black Phosphorus Nanosheets for Sequential Thrombolysis and Reactive Oxygen Species Scavenging in Ischemic Stroke Treatment. Biomater. Sci..

[33] Xu J., Shi C., Ding Y., Qin T., Li C., Yuan F., Liu Y., Xie Y., Qin Y., Cao Y. (2024). Endothelial Foxo1 Phosphorylation Inhibition via Aptamer-Liposome Alleviates OPN-Induced Pathological Vascular Remodeling Following Spinal Cord Injury. Adv. Sci..

[34] Yu T., Wei Z., Wang J., Song C., Huang W., Zhang P., Shi J., Zhang R., Jiang M., Wang D. (2025). Ginkgo Biloba Extract GBE50 Ameliorates Cerebrovascular Dysfunction and Cognitive Impairment in a Mouse Model of Alzheimer’s Disease. Phytomedicine.

[35] Wang J.-L., Huang Q.-M., Hu D.-X., Zhang W.-J. (2024). Therapeutic Effect of Exosomes Derived from Schwann Cells in the Repair of Peripheral Nerve Injury. Life Sci..

[36] Lu H., Li S., Dai D., Zhang Q., Min Z., Yang C., Sun S., Ye L., Teng C., Cao X. (2022). Enhanced Treatment of Cerebral Ischemia-Reperfusion Injury by Intelligent Nanocarriers through the Regulation of Neurovascular Units. Acta Biomater..

[37] Zhang Q., Li S., Chen H., Yin J., Chen Y., Liu L., He W., Min Z., Gong Y., Xu J. (2024). Reduction of Oxidative Stress and Excitotoxicity by Mesenchymal Stem Cell Biomimetic Co-Delivery System for Cerebral Ischemia-Reperfusion Injury Treatment. Small.

[38] Schirone L., Forte M., D’Ambrosio L., Valenti V., Vecchio D., Schiavon S., Spinosa G., Sarto G., Petrozza V., Frati G. (2022). An Overview of the Molecular Mechanisms Associated with Myocardial Ischemic Injury: State of the Art and Translational Perspectives. Cells.

[39] Allaire M., Al Sayegh R., Mabire M., Hammoutene A., Siebert M., Caër C., Cadoux M., Wan J., Habib A., Le Gall M. (2023). Monoacylglycerol Lipase Reprograms Hepatocytes and Macrophages to Promote Liver Regeneration. JHEP Rep..

[40] Kespohl M., Goetzke C.C., Althof N., Bredow C., Kelm N., Pinkert S., Bukur T., Bukur V., Grunz K., Kaur D. (2024). TF-FVIIa PAR2-β-Arrestin Signaling Sustains Organ Dysfunction in Coxsackievirus B3 Infection of Mice. Arterioscler. Thromb. Vasc. Biol..

[41] Zhong T., Cao Q., Ma Z., Jiang C. (2025). Metabolic Regulation of Interferon-Mediated Innate Antiviral Immunity. Front. Immunol..

[42] Li Q., Qin X., Wang L., Hu D., Liao R., Yu H., Wu Z., Liu Y. (2025). Multi-Time Point Transcriptomics and Metabolomics Reveal Key Transcription and Metabolic Features of Hepatic Ischemia-Reperfusion Injury in Mice. Genes Dis..

[43] Gao F., Qiu X., Wang K., Shao C., Jin W., Zhang Z., Xu X. (2022). Targeting the Hepatic Microenvironment to Improve Ischemia/Reperfusion Injury: New Insights into the Immune and Metabolic Compartments. Aging Dis..

[44] Triantafyllou G.A., Triantafyllou A., Zafeiridis A.S., Koletsos N., Zafeiridis A., Gkaliagkousi E., Douma S., Dipla K. (2022). Association of cerebral oxygenation during exercise with target organ damage in Middle-Aged hypertensive and normotensive individuals. Am. J. Hypertens..

[45] Kaltenmeier C., Wang R., Popp B., Geller D., Tohme S., Yazdani H.O. (2022). Role of Immuno-Inflammatory Signals in Liver Ischemia-Reperfusion Injury. Cells.

[46] Shi G., Zhang Z., Ma S., Li Y., Du S., Chu Y., Li Y., Tang X., Yang Y., Chen Z. (2021). Hepatic Interferon Regulatory Factor 8 Expression Mediates Liver Ischemia/Reperfusion Injury in Mice. Biochem. Pharmacol..

[47] Dossi C.G., Vargas R.G., Valenzuela R., Videla L.A. (2021). Beneficial Effects of Natural Compounds on Experimental Liver Ischemia-Reperfusion Injury. Food Funct..

[48] George J., Lu Y., Tsuchishima M., Tsutsumi M. (2024). Cellular and Molecular Mechanisms of Hepatic Ischemia-Reperfusion Injury: The Role of Oxidative Stress and Therapeutic Approaches. Redox Biol..

[49] Wu D., Wang S., Wang F., Zhang Q., Zhang Z., Li X. (2024). Lactate Dehydrogenase A (LDHA)-Mediated Lactate Generation Promotes Pulmonary Vascular Remodeling in Pulmonary Hypertension. J. Transl. Med..

[50] Yoval-Sánchez B., Ansari F., James J., Niatsetskaya Z., Sosunov S., Filipenko P., Tikhonova I.G., Ten V., Wittig I., Rafikov R. (2022). Redox-Dependent Loss of Flavin by Mitochondria Complex I Is Different in Brain and Heart. Redox Biol..

[51] Hansen L.M.B., Dam V.S., Guldbrandsen H.Ø., Staehr C., Pedersen T.M., Kalucka J.M., Beck H.C., Postnov D.D., Lin L., Matchkov V.V. (2025). Spatial Transcriptomics and *Proteomics* Profiling after Ischemic Stroke Reperfusion: Insights into Vascular Alterations. Stroke.

[52] Movahed M., Brockie S., Hong J., Fehlings M.G. (2021). Transcriptomic Hallmarks of Ischemia-Reperfusion Injury. Cells.

