# Supplementary material for: Molecular Dissection of Permanent vs. Reperfused Ischemia: Multi-Omics Divergence and Precision Therapeutic Implications
Source: Curr Issues Mol Biol. 2026 Jan 22;48(1):124. doi: 10.3390/cimb48010124 (PMC12840552; doi:10.3390/cimb48010124)
Supplement: Supplementary file 1 [file cimb-48-00124-s001.zip › Supplementary figures.pdf]

Molecular Dissection of Permanent vs Reperfused Ischemia: Multi-omics Divergence and Precision Therapeutic Implications

Supplementary summary

Pathway enrichment analysis of common and condition-specific differentially expressed genes (DEGs) revealed distinct biological signatures. Common DEGs (n=2,036) were strongly enriched in inflammatory and innate immune pathways, including cytokine signaling and type I interferon responses, reflecting a core molecular stress response to ischemia. IR-specific DEGs (n=1,767) were associated with cellular stress responses, endoplasmic reticulum/unfolded protein response, RNA processing, and myelination-related processes, highlighting pathways of cellular dysfunction and damage in untreated ischemia. In contrast, IRI-specific DEGs (n=600) were enriched in neuronal development, axon guidance, calcium signaling, and synaptic transmission, suggesting that the intervention promotes mechanisms related to neuronal repair, network reorganization, and functional recovery. Together, these findings indicate that the intervention shifts the molecular landscape from injury-associated stress pathways toward those involved in neural repair and homeostasis.

Supplementary figures and legends:

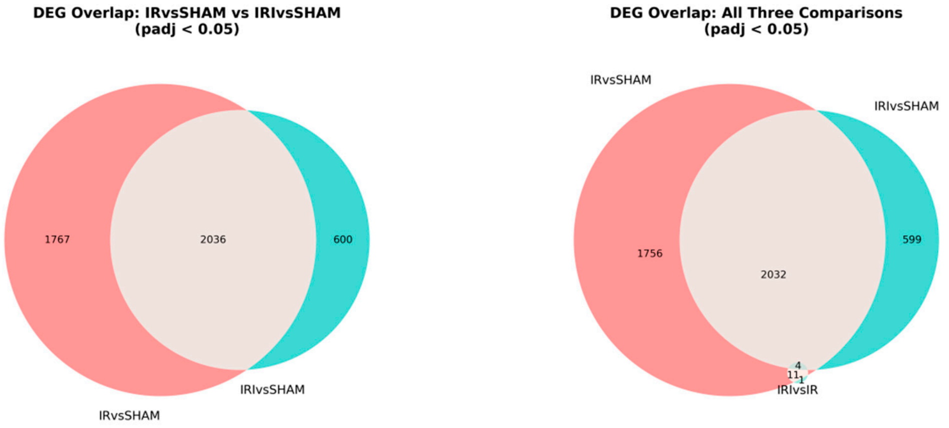

Figure S1. Venn diagram of overlapping differentially expressed genes (DEGs) between experimental comparisons. DEGs (padj < 0.05) identified in the three contrasts—IRvsSHAM, IRIvsSHAM, and IRIvsIR—are shown, with numbers indicating the count of genes in each unique and intersecting set. The largest overlap (2036 genes) is shared between the IRvsSHAM and IRIvsSHAM comparisons, highlighting common transcriptional responses to ischemia.

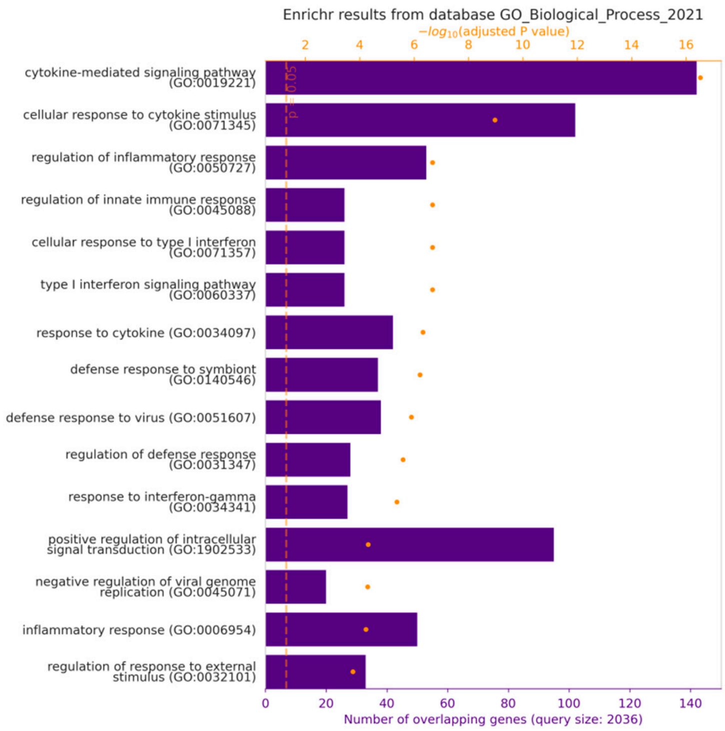

Figure S2: Pathway enrichment analysis of common DEGs shared between IR and IRI conditions. Bar plot shows the top 15 significantly enriched Gene Ontology (GO) Biological Process terms for the 2,036 genes differentially expressed in both IRvsSHAM and IRIvsSHAM comparisons. The x-axis represents the -$\log_{10}$(adjusted P-value); the y-axis lists the enriched pathways. Bars are colored by the number of overlapping genes. The analysis highlights strong enrichment in immune and inflammatory response pathways, indicating a core ischemic stress signature

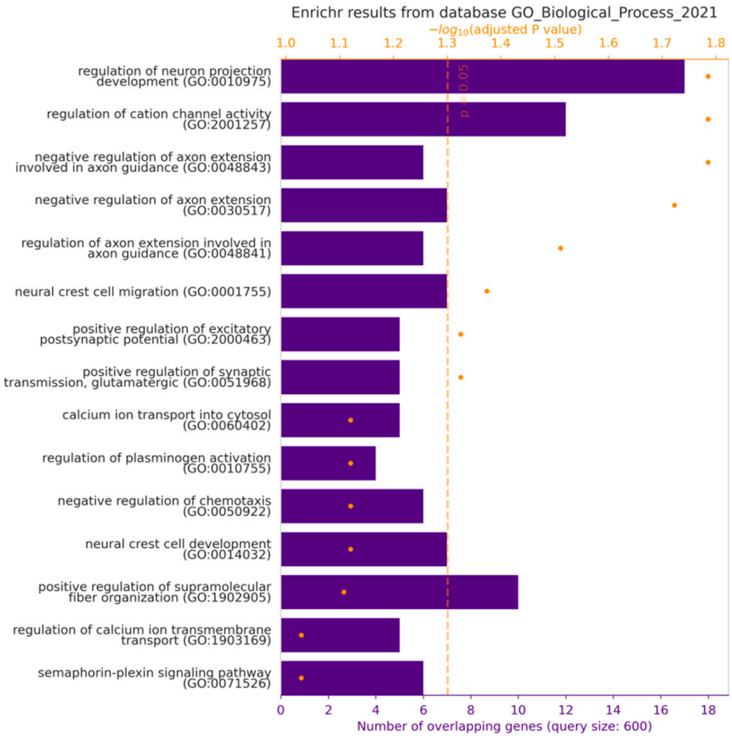

Figure S3: Pathway enrichment analysis of IRIvsSHAM-specific DEGs. Bar plot illustrates the top 15 significantly enriched GO Biological Process terms for the 600 genes uniquely differentially expressed in the IRIvsSHAM comparison. The x-axis represents the -$\log_{10}$(adjusted P-value). Enriched pathways are related to neuron projection development, cation channel activity, axon guidance, synaptic transmission, and neural crest cell migration, suggesting intervention-mediated engagement of neurorepair and signaling modulation mechanisms.

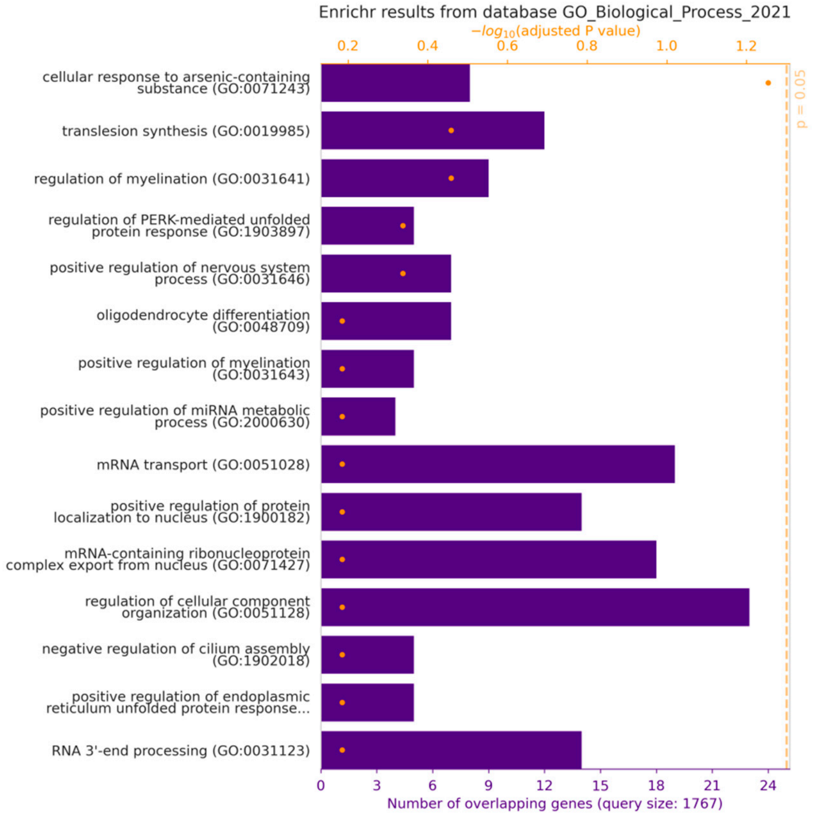

Figure S4: Pathway enrichment analysis of IRvsSHAM-specific DEGs. Bar plot displays the top 15 significantly enriched GO Biological Process terms for the 1,767 genes uniquely differentially expressed in the IRvsSHAM comparison. The x-axis represents the -$\log_{10}$(adjusted P-value). Enriched terms are associated with cellular stress responses (e.g., response to arsenic, translesion synthesis), regulation of myelination, ER stress, and RNA processing, reflecting pathways activated in untreated ischemic injury.

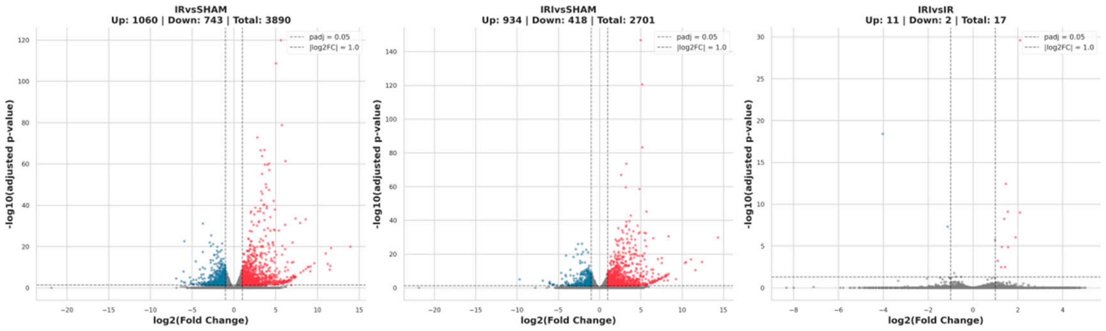

Figure S5. Volcano plots visualizing differential expression for each comparison. Each plot displays genes according to their $\log_2$ fold change (x-axis) and statistical significance (–$\log_{10}$ adjusted p-value; y-axis). Significantly up- and down-regulated genes (padj < 0.05 and $|\log_2 FC| > 1$) are highlighted in red and blue, respectively. Total numbers of significant DEGs are shown above each plot: (A) IRvsSHAM (1060 up, 743 down), (B) IRIvsSHAM (934 up, 418 down), and (C) IRIvsIR (11 up, 2 down).
