# Supplementary material for: Molecular Dissection of Permanent vs. Reperfused Ischemia: Multi-Omics Divergence and Precision Therapeutic Implications
Source: Curr Issues Mol Biol. 2026 Jan 22;48(1):124. doi: 10.3390/cimb48010124 (PMC12840552; doi:10.3390/cimb48010124)
Supplement: Supplementary file 1 [file cimb-48-00124-s001.zip › Supplementary table.pdf]

Supplementary Table S1. Animal behavior evaluation of cerebral ischemia

**score**	**Behavioral performance**	nerve injury disorder
0	No nerve injury disorder	NO
1	Can't fully extend the opposite front paw	Yes
2	Turn around to the paralyzed side	Yes
3	Tilt to the opposite side	Yes
4	Unable to walk spontaneously, loss of consciousness	Yes
